# Postmating Female Control: 20 Years of Cryptic Female Choice

**DOI:** 10.1016/j.tree.2017.02.010

**Published:** 2017-05

**Authors:** Renée C. Firman, Clelia Gasparini, Mollie K. Manier, Tommaso Pizzari

**Affiliations:** 1 Centre for Evolutionary Biology, School of Biological Sciences, University of Western Australia, 35 Stirling Hwy, Perth, Western Australia, 6009, Australia; 2 Biological Sciences, The George Washington University, 800 22nd St. NW Suite 6000, Washington, DC 20052, USA; 3 Department of Zoology, Edward Grey Institute, University of Oxford, Oxford, OX1 3PS, UK

## Abstract

Cryptic female choice (CFC) represents postmating intersexual selection arising from female-driven mechanisms at or after mating that bias sperm use and impact male paternity share. Although biologists began to study CFC relatively late, largely spurred by Eberhard’s book published 20 years ago, the field has grown rapidly since then. Here, we review empirical progress to show that numerous female processes offer potential for CFC, from mating through to fertilization, although seldom has CFC been clearly demonstrated. We then evaluate functional implications, and argue that, under some conditions, CFC might have repercussions for female fitness, sexual conflict, and intersexual coevolution, with ramifications for related evolutionary phenomena, such as speciation. We conclude by identifying directions for future research in this rapidly growing field.

## Trends

In 1996, Eberhard crystallized the idea of CFC as an engine of sexual selection and initiated the study of female-driven processes.

Demonstrating CFC, which is defined as female-mediated morphological, behavioral, or physiological mechanisms that operate to bias fertilization toward the sperm of specific male(s), requires dissecting male and female variance components of sperm retention or paternity.

Technologies developed over the past 20 years have helped elucidate the proximate mechanisms underpinning fertilization and have accelerated the field of CFC.

Females may bias sperm use at successive stages of the reproductive process, including shortly after mating, during sperm transit and/or storage, and at fertilization.

CFC can have fundamental repercussions for sexual selection on males, female fitness, and, consequently, sexual conflict and intersexual coevolution, with ramifications for related evolutionary phenomena (e.g., speciation).

## The Last Piece of Darwin’s Puzzle

**Figure 1:**
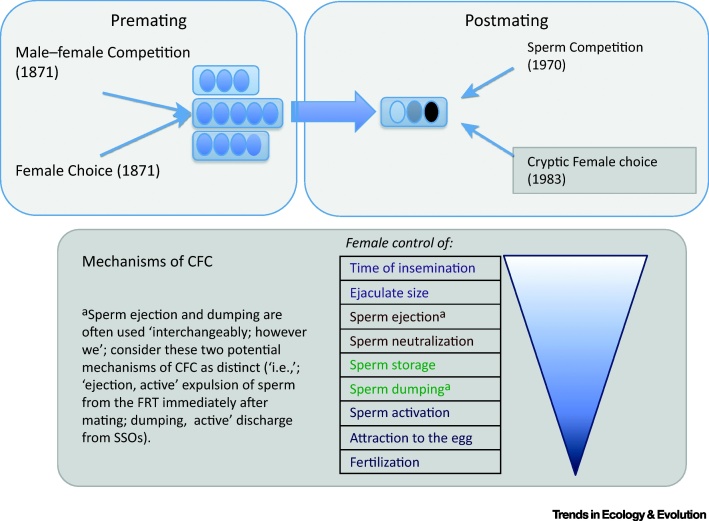
Sexual Selection by Cryptic Female Choice (CFC). (A) CFC is a postmating episode of intersexual selection on males. Premating episodes of sexual selection, as they were formalized by Darwin in the *Descent of Man*[1], act on variance in male access to different females and their eggs (small ovals grouped into separate clutches). Polyandry creates potential for postmating sexual selection acting on variation in the paternity of the eggs within each clutch (represented by eggs of different colors). Sperm competition was recognized by Parker in 1970 [2], while CFC was identified as an engine of sexual selection in 1983 [4]. Several factors can explain why CFC was only appreciated so late. First, postmating mechanisms are inherently obscure and hard to study; often, they are mediated by subtle molecular interactions and in internal fertilizers occur within the female reproductive tract (FRT). Male-driven mechanisms, such as the number of sperm inseminated, appear, at least superficially, more obvious than patterns of differential sperm utilization by females. Finally, an inherent male-dominated cultural bias likely predisposed researchers to male-driven explanations of postmating patterns, reminiscent of the skepticism that met Darwin’s idea of premating female choice a century earlier. (B) Postmating processes through which females can control competitive fertilization success after mating (listed in approximate order of occurrence during and after mating; color coded: at mating, shortly following insemination, over prolonged sperm storage, around the time of fertilization). We discuss empirical evidence of these mechanisms in the main text, and restrict our focus to prezygotic stages, excluding mechanisms of differential abortion and maternal investment, which influence offspring fitness rather than paternity share. The arrow on the right represents the proportion of the ejaculate neutralized at successive stages. Mechanisms closer to fertilization deal with fewer sperm and, consequently, must be more precise than mechanisms acting at earlier stages. Some of these mechanisms are more relevant to internal fertilizers than to other organisms (e.g., sessile broadcast spawners). Abbreviation: SSO, sperm storage organ.

Darwin’s exposition on **sexual selection** (see Glossary) was restricted to premating episodes in internal fertilizers; for males, these episodes comprised intrasexual competition to access receptive females, and intersexual selection exerted by females discriminating among prospective partners [1]. Approximately one century later, Geoff Parker intuited that intrasexual selection can continue after mating, because widespread **polyandry** leads to **sperm competition** [2]. This realization raised the possibility that intersexual selection also occurs during and after mating if polyandrous females can bias sperm utilization [3], a process that Randy Thornhill called ‘cryptic female choice’ (CFC) [4] (Figure 1A). In 1996, Bill Eberhard crystallized the idea of CFC as an engine of sexual selection in his book *Female Control: Sexual Selection by Cryptic Female Choice* [5], elaborating on Thornhill’s initial definition of CFC as female-mediated morphological, behavioral, or physiological mechanisms that bias fertilization toward the sperm of specific males. Eberhard was instrumental in extending postmating sexual selection to the study of female-driven processes [5]. Molecular tools, along with *in vitro* and *in vivo* technologies developed over the past 20 years, have helped elucidate the proximate mechanisms underpinning fertilization and, combined with the increasing appreciation for female roles in sexual selection, have accelerated the study of CFC [6].

Here, we appraise progress in the field 20 years after the publication of Eberhard’s pivotal book. We distinguish between proximate mechanisms and functional implications of CFC. First, we explain criteria for demonstrating CFC, outline proximate mechanisms underpinning CFC, critically review empirical evidence, and detail current approaches for resolving these mechanisms. We then investigate functional implications of CFC, and discuss its evolutionary significance for females, males, and intersexual coevolution. Wherever possible, we include examples that represent clear demonstrations of CFC and associated fitness consequences, and we also speculate about potential or hypothetical examples of CFC. While a comprehensive survey of the literature is beyond the scope of this review, we hope to encourage discussion and further research in areas where unequivocal evidence is still lacking.

## Demonstrating CFC

CFC is mediated by subtle and complex processes, and often comprises covert mechanisms that are within the female reproductive tract (FRT), which historically have proven technically difficult to study. Measuring CFC is further complicated by the necessary co-occurrence of sperm competition. To demonstrate CFC, we need to: (i) identify a female trait or behavior that affects sperm uptake and/or utilization at or after mating; and (ii) show that this female response is differential and nonrandom, such that the sperm of certain males are predictably favored or disfavored based on factors such as phenotype or genotype. Box 1 outlines a general quantitative framework to test CFC; below, we review recent empirical approaches addressing (i) and (ii).

Box 1 **Defining and Demonstrating CFC**

CFC is operationally defined as variation in fertilization success among males due to nonrandom, differential responses of females; thus, demonstrating CFC requires dissecting male and female variance components of sperm retention or paternity. Although demonstrating CFC has been historically debated [84], the approach outlined below is now widely accepted. The simplest case is a factorial design where females are exposed to sperm of individual males to distinguish consistent patterns of sperm utilization from random error. Each male–female combination is replicated using the same or genetically similar individuals (e.g., full-sibs, isogenic, or inbred lines). We partition Sum of Squares within (*SS_within_*, error) and between (*SS_between_*) male–female combinations; *SS_between_* is then partitioned across the male and female main effects and their male × female interaction. When *SS_within_* >SS_between_, variation is random across male–female combinations, while *SS_within_ <SS_between_* indicates significant differences. A good example of this general approach is provided by a study of *Drosophila melanogaster* [85], in which the repeated use of individual males with individual females enabled the authors to estimate the repeatability of P_1_ and P_2_. A significant female effect indicates consistent differences among females in sperm utilization (e.g., they might lose sperm from SSO at faster rate), regardless of male identity. This scenario can have interesting repercussions for sexual selection on males if males mate nonrandomly with respect to female type, but does not in itself represent CFC. A significant male effect indicates consistent variation among males independent of female identity due to either male effects (e.g., variation in ejaculate fertilizing efficiency) or directional CFC for certain male traits. The two alternatives are not mutually exclusive, and special care is required to distinguish male and female mechanisms. One approach is to measure ejaculate phenotypes related to competitive fertilization success (e.g., sperm numbers or velocity) and generate expectations of paternity share based on the relative values of these male traits. Deviations from such expectations are inconsistent with sperm competition explanations and instead lend support to CFC. For example, Parker *et al.* [86] generated expectations for P_2_ based on S_2_, and this approach was later modified for non-normal data 87, 88 and multiple SSOs [89]. Finally, a significant male × female interaction indicates nondirectional CFC, consistent differences across male–female combinations in utilization or fertilization success [90].

This approach can be expanded to include sperm competition between two males and attributing variation in P_2_ to the female, first male, second male, or male × male and female × male × male interactions. We can also test hypotheses that certain factors influence CFC by including male or female genotype or phenotype as a main or random effect, depending on experimental design. In the case of directional CFC on a continuous variable, we can use selection analysis to express male fitness (fertilization success, *W*) as a function of the male phenotype, *z*, targeted by CFC (Equation I):

[I], W=β(z)+ε

where *ε* is an error term, *W* and *z* represent standardized male fitness and phenotype, respectively, and *β* represents the standardized gradient of postmating intersexual selection on z (i.e., *β* = *S/σ_p_*, where *S* is the CFC selection differential). However, the causal relationship between male trait and female response can only be demonstrated through experimental manipulations.

Alt-text: Box 1

### Experimental Manipulation of Male Quality and Compatibility

The causal relationship between a male trait and patterns of female sperm utilization can be illuminated by experimentally manipulating male phenotype while controlling for, or blocking, other factors. For context-dependent phenotypes, such as social status or relatedness, a powerful design involves allowing females to evaluate the same male in different contexts. Changes in female sperm utilization and/or fertilization success associated with such manipulations are consistent with CFC (e.g., [7]). However, plastic male responses (e.g., differential sperm allocation) must be controlled for, increasing the difficulty of demonstrating CFC. Artificial insemination (AI) or *in vitro* assays of sperm utilization and fertilization can be used to control ejaculate traits and eliminate the influence of premating mechanisms (e.g., 8, 9, 10, 11). A limitation of *in vitro* approaches is that they can remove some CFC mechanisms triggered by female assessment of male phenotype. However, AI can be used to experimentally manipulate female perception, such as by exposing a female to one male while inseminating her with the sperm of another [12].

### Differentiating Sperm of Different Males

Distinguishing sperm from different males presents a challenge to understanding postmating mechanisms. One solution uses among-male variation in sperm traits to test differential positioning in the female sperm storage organ (SSO) [13] or fertilization success [14]. Competitive PCR of microsatellites has been used to quantify **S_2_** for individual males within the SSOs of multiply mated females 15, 16. Differential labeling of sperm from multiple males has allowed high-resolution characterization of postmating mechanisms, including those related to CFC 8, 9, 17. The recent development of transgenic males producing live sperm expressing green or red fluorescent proteins has enabled unprecedented insights into the behavior of sperm within the FRT, and CFC mechanisms 18, 19, 20, 21.

## Potential Mechanisms

Eberhard identified multiple proximate mechanisms through which females might bias fertilization at successive stages of the reproductive process [5]. Here, we focus on prezygotic mechanisms at and shortly after mating, mediating sperm storage in the SSO, and at fertilization (Figure 1B).

### Differential Responses at and Shortly after Mating

Females might first influence paternity by controlling the timing and order of competing inseminations. Females of the moth *Ephestia kuehniella* influence **P_2_** by remating sooner, through displacement of the first **spermatophore** from the SSO [22]. Moreover, the outcome of sperm competition is often mediated by the number of sperm inseminated by different males. While ejaculate size is largely under male control, females might influence sperm transfer through spermatophore acceptance or by actively terminating copulation. An elegant study in the guppy *Poecilia reticulata* showed that a male inseminates more sperm if his mate perceives him to be relatively attractive [7]. Although poorly investigated, female control over copulation duration represents an effective mechanism for mediating which sperm enter the fertilizing pool 23, 24

**Figure 2:**
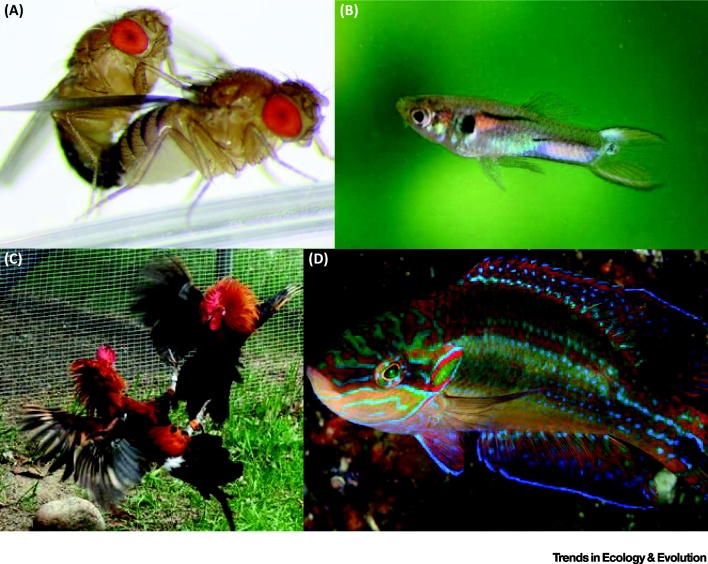
Examples of Directional Cryptic Female Choice (CFC) for Male Phenotype. (A) Mating *Drosophila melanogaster*. Selection experiments in *D. melanogaster* and new comparative evidence across *Drosophila* species indicate that directional CFC targets sperm size, promoting the evolution of giant sperm, one of the most exaggerated sexual ornaments [54]. This appears to be the result of a Fisherian-like process in which female seminal receptacles (SR) length is genetically correlated with sperm length as well as with ejection time, remating rate, and sperm displacement [54]. (B) Male coloration in guppies, *Poecilia reticulata*. Female guppies prefer to mate with more colorful males, particularly those sporting a relatively large carotenoid-based patch. Pilastro *et al.*[7] demonstrated a role of CFC by manipulating the perception of male attractiveness to females, who actively favored fertilization by brightly colored males, controlling the duration of the copula and, thus, the number of sperm transferred [24]. Females terminated copulation earlier and received fewer sperm with males that were perceived of lower quality through the comparison with another more colorful male [7]. (C) Male feral fowl, *Gallus domesticus*, competing for social status. Male social dominance appears to be favored by CFC in some populations. Females can eject ejaculates immediately following insemination, when approximately 89% is expelled on average 28, 29. Females of a feral population were found to vary predictably in the probability (risk) of sperm ejection and the proportion of ejaculate lost (intensity). Part of this variation is explained by mechanical properties, for example, larger ejaculates suffer a higher ejection risk, possibly because it is harder for females to uptake these inseminations given the lack of intromission. However, other patterns suggest differential sperm ejection by females (e.g., risk increases as females accumulate successive matings and control for ejaculate volume; thus, socially subordinate males suffer higher ejection intensity 28, 29. (D) Nesting male ocellated wrasse, *Symphodus ocellatus*. In this externally fertilizing species, CFC favors fertilization by ‘nesting' males. Males adopt alternative mating tactics: ‘nesting’ males attend nests where females lay their eggs, while ‘sneaker' and ‘satellite' males scrounge fertilizations by visiting the nests of nesting males. Nesting males produce faster sperm, while sneakers produce more sperm. Recent experimental evidence demonstrates that female ovarian fluid (OF) biases sperm competition dynamics to increase the relative importance of sperm velocity over sperm numbers, thus favoring the ejaculates of nesting males and reinforcing female premating preference for these males [44]. Reproduced, with permission, from Amy Hong (A), C. Gasparini (B), and H. Løvlie (C).

In several species, a proportion of the ejaculate is lost shortly following ejaculation and female processes, such as differential sperm ejection, digestion, and incapacitation, influence which sperm are retained. In some invertebrates, differential sperm ejection is associated with male size 21, 25, species identity [26], and courtship duration [27]. Similarly, sperm ejection by female feral fowl *Gallus domesticus* might disfavor inseminations by socially subdominant males 28, 29 (Figure 2). Female kittiwakes *Rissa tridactyla* can utilize sperm ejection to reduce the risk of fertilization by sperm aging within the FRT from previous copulations, which compromises offspring viability [30]. Sperm ejection might be male induced in the socially polyandrous dunnock *Prunella modularis*, where the male pecks the female cloaca before mating, which stimulates ejection of previously stored semen from other males [31], although the extent to which males control this female response remains unclear.

Mechanisms of sperm uptake can also create opportunities for CFC, such as contractions of the FRT that facilitate sperm passage from lower to upper FRT in red garter snakes *Thamnophis sirtalis parietalis* [32]. In some primates, the degree of sperm uptake has been linked to contractions associated with female orgasm, and female Japanese macaques *Macaca fuscata* are more likely to achieve orgasm-like responses when mating with socially dominant males [33], suggesting preferential sperm uptake for these males. Finally, sperm might be attacked by innate or acquired immune responses, phagocytosed, digested, or incapacitated within the FRT, such as by spermicidal action (e.g., *Drosophila pseudoobscura* [34]). Females might also exert their control by alleviating sperm incapacitation by rival ejaculates (e.g., bees and ants [35]).

Out of all of these prestorage female-mediated phenomena, evidence that they function as CFC appears clearer for differential sperm ejection in relation to male phenotypes, although even here the causal effect of female response on paternity share remains largely unresolved.

### Differential Sperm Storage

If sperm reach storage having escaped ejection, digestion, or incapacitation, they might interact with sperm from other males through displacement, stratification, or mixing. Eberhard first suggested that FRT complexity can increase female control over sperm storage and paternity [5]. Indeed, SSO morphology can influence the degree to which sperm are stored and/or displaced. Female dung flies *Scathophaga stercoraria* with four SSOs might be better able to control paternity compared with females with only three SSOs [36]. In *Drosophila melanogaster*, longer sperm are favored when stored in longer female seminal receptacles (SR) [37] due to their superior ability to displace, and resist displacement by, shorter sperm [38], exemplifying that mechanisms of CFC and sperm competition are not mutually exclusive and often work through a process of male–female interaction. Once stored, sperm can be lost from the SSOs in a process referred to as sperm ‘dumping’ [39] (Figure 1B). Dumping has been suggested to occur in several invertebrate taxa (e.g., [40]).

### Selective Fertilization

#### Differential Mediation of Sperm Performance

As a key determinant of fertilizing efficiency, sperm swimming performance offers an important mechanism through which females can bias fertilization. **Female reproductive fluids** are emerging as widespread modulators of sperm swimming. Differential **sperm chemotaxis** was first demonstrated in a mussel *Mytilus galloprovincialis*, where chemoattractants in the fluid associated with the eggs differentially mediate the migration of sperm of individual males by changing sperm swimming behavior [41]. Sperm swimming velocity is determined by the interaction between the identity of the male sperm donor and the female ‘ovarian fluid’ (OF) donor in several external fertilizers (e.g., 42, 43). In the externally fertilizing ocellated wrasse *Symphodus ocellatus*, OF provides a mechanism by which females can bias the outcome of fertilization toward certain ‘nesting’ male phenotypes [44] (Figure 2), while, in the guppy, an internal fertilizer, *in vitro* evidence indicates that OF mediates sperm swimming velocity to bias paternity toward unrelated males [10].

FRT secretions can also mediate differential sperm activation, where sperm must undergo postmating transformations to achieve fertilization. In spiders, secretions from the SSO break sperm capsules to release them, creating the opportunity for females to selectively activate the sperm of different males [45]. In mammals, the pivotal role that FRT secretions have in sperm capacitation and hyperactivation might enable discrimination among sperm of rival males [46]. The differential effects of reproductive fluids offer significant potential for CFC.

#### Sperm–Egg Signaling

There is some evidence, largely from *in vitro* studies, that CFC can occur during sperm–egg interactions. External fertilization in sea urchins *Echinometra mathaei* and *Echinometra oblonga* is mediated by the sperm protein bindin, which is highly polymorphic within species [47]. This variation leads to assortative fertilization: in situations where all males have an equal opportunity to fertilize eggs, female sea urchins produce eggs that nonrandomly select sperm with a *bindin* genotype similar to their own [47]. The sea urchin egg glycoprotein EBR1 might facilitate gamete fusion by targeting sperm bindin through cell surface signaling [48]. Egg glycoproteins appear to have a similar function in house mice *Mus musculus domesticus*; a mismatch with sperm surface proteins leads to a significant reduction in litter size [49]. Gamete protein signaling in this species might also account for egg selection of specific sperm genotypes to avoid inbreeding [9] or to promote certain major histocompatibility complex (MHC) haplotypes [50] (Box 2).

Box 2 **CFC and the Vertebrate MHC**

The vertebrate major histocompatibility complex (MHC) is a highly polymorphic haplotype that primarily functions in immune regulation but also as a genetic compatibility system [91]. An individual’s ability to respond to pathogens has been linked to MHC polymorphism, with a number of evolutionary mechanisms contributing to the maintenance of high levels of MHC variation [91]. In many species, MHC genes play a critical role in self-nonself recognition, and appear to be involved in female mate choice, for example, through olfactory cues [91]. Given that MHC genes are critical for immune function, an increase in MHC heterozygosity, or the procurement of rare alleles within the MHC complex, is expected to lead to increased resistance among offspring. Consequently, mechanisms of CFC might be expected to favor the sperm of either dissimilar males or males with ‘optimal’ MHC similarity [91]. MHC-based disassortative fertilization might be a strategy to prevent inbreeding or maximize general genomic heterozygosity (enabling a wider recognition of pathogens; ‘heterozygote advantage’), leading to increased offspring fitness [91]. The parasite Red Queen hypothesis posits that, when new combinations of genes are required to provide the best immune response in each generation, female choice for resistance genes that complement their own MHC genotype could, in theory, drive MHC diversity [91]. In the stickleback, individuals with an intermediate number of MHC alleles suffer lower levels of parasite infection, suggesting that MHC heterozygosity is optimized, rather than maximized, through female choice [91]. After mating, this process can occur via mechanisms of CFC that bias fertilizations toward sperm with complementary alleles [91].

MHC-dependent gamete fusion has been demonstrated in different taxa (mice [50], salmon [92], and guppies [93]), but what is the specific mechanism driving MHC-based sperm selection? Contradictory reports on whether sperm signal their MHC haplotype suggest that expression might depend upon male infection status [50]. Strong linkage disequilibrium between testis-expressed MHC genes and MHC-linked olfactory receptor genes in some taxa could indicate which MHC alleles are carried by the sperm (the sperm receptor selection hypothesis [91]). The complexity involved with MHC-based sperm selection is apparent in the red junglefowl, in which females might use premating phenotypic cues to select MHC-dissimilar sperm and avoid fertilizations by related males [11]. This system requires that females ‘know’ their own MHC genes and be able to assess those of their partners both before and after mating. Clearly, more research is required to precisely establish the mechanisms explaining MHC-based CFC.

Alt-text: Box 2

In sea urchins and mice, ‘**egg defensiveness**’, a possible adaptation to the risk of pathological polyspermy, might also function as a means of filtering and selecting sperm that are compatible with the egg, or are of sufficient quality 8, 51, 52, 53. Similarly, variation in the number or density of cells associated with the cumulus oophorus in mammals is a potential barrier by which females can control fertilization rates under different risks of pathological **polyspermy** [53]. Both within and between *Mus* species, the degree of gamete incompatibility is positively associated with sperm competition level, suggesting that the ‘discriminatory’ nature of eggs becomes greater as the intensity of postmating sexual selection increases 8, 52. Convergent patterns observed among internal and external fertilizers suggest that CFC mechanisms mediating sperm-egg fusion are a phylogenetically widespread phenomenon.

## Evolutionary and Functional Implications of CFC

### Functional Significance for Females

Nonrandom sperm utilization requires adaptive explanations, and several have been proposed over the past 20 years (Table 1). Resolving the adaptive significance of CFC for females is intrinsically tied to the adaptive significance of premating female choice and polyandry. As in premating female choice, adaptive explanations of CFC fall into two broad categories: (i) CFC is adaptive to females and has evolved specifically for the fitness benefits that controlling sperm utilization conveys to females; and (ii) CFC is not adaptive to females and represents either a side effect of other adaptive female traits (e.g., sensory bias) and/or male manipulation of female sperm utilization (e.g., males inducing females to bias sperm utilization in favor of their ejaculates even when this is against the fitness interest of the female; Table 1). Most explanations fall under (i), where CFC is seen as a means for polyandrous females to control paternity when premating choice is difficult or otherwise constrained. A key difference with premating female choice is that the fitness benefits of CFC are likely to occur exclusively through increased offspring fitness (i.e., genetic benefits). These genetic benefits may be shared across females (generating **directional selection**), or females may vary in their preferred criteria (generating **nondirectional selection**). In the former scenario, females might favor fertilization by males of certain phenotypes. Genetic mechanisms of **good genes** and **Fisherian runaways** are often invoked to explain the evolution of directional CFC (e.g., [54]; Figure 2A). Under the **good sperm hypothesis** [55], the postmating offshoot of the good genes hypothesis, polyandry selects for ejaculate traits correlated with male genetic quality. The related **sexy sperm hypothesis** [56] predicts that males successful in sperm competition sire sons with superior ejaculate traits. Both models require a genetic correlation between intrinsic sperm competitiveness and either genetic quality (good sperm) or female polyandry (sexy sperm). While neither model assumes a female role beyond mating multiply, CFC could catalyze both mechanisms.

**Table 1:** Adaptive Explanations of CFC.^a^

Mechanism	Evidence	Possible outcomes and patterns ofselection on males	Refs
Side effect of adaptive female traits			
Consequence of obtaining nutrients through male courtship feeding	Scorpionfly *Harpobittacus nigriceps*	Directional	[4]
Consequence of egg stimuli for oviposition	Damselfly *Calopteryx haemorrhoidalis asturica*	Directional	[80]
Consequence of immunological defense against pathogens	NA	Nondirectional	
Defense against polyspermy	Sea urchins *Strongylocentrotus purpuratus, Strongylocentrotus franciscanus,* and *Strongylocentrotus droebachiensis*	Likely nondirectional	[51]
	House mouse *Mus musculus*		8, 52, 53
Increased reproductive success			
Increased offspring viability (good genes)	Brown antechinus *Antechinus stuartii*	Directional	[81]
Increased offspring reproductive success (Fisherian process)	Fruit flies *Drosophila* spp.	Directional	[54]
Optimal sex allocation	Brown anole *Anolis sagrei*	Possible selection on male phenotype through sex-biased offspring production	[57]
Increased genetic diversity of the whole brood	NA	Nondirectional	
Inbreeding avoidance	Cricket *Teleogryllus oceanicus*	Nondirectional disassortative fertilization	12, 16, 60
	Field cricket *Gryllus bimaculatus*		15, 60
	Orb-web spider *Argiope lobata*		[62]
	Guppy *Poecilia reticulata*		[10]
	Red junglefowl *Gallus gallus*		[11]
	House mouse *M. musculus*		[9]
Hybridization avoidance	Fruit flies *Drosophila simulans* × *Drosophila mauritiana*	Nondirectional assortative fertilization	[27]
	Crickets *Gryllus bimaculatus* × *Gryllus campestris*		[82]
	Atlantic salmon *Salmo salar* × Brown trout *Salmo trutta*		[83]
Genetic compatibility	Mussel *Mytilus galloprovincialis*	Nondirectional	[41]
Offspring heterozygosity	Chinook salmon *Oncorhynchus tshawytscha*	Nondirectional disassortative fertilization	[43]
Offspring homozygosity	Dungfly *Scathophaga stercoraria*	Disruptive assortative fertilization	[36]
Red Queen	NA	Frequency dependent	

a The table details different categories of potential fitness benefits to females associated with CFC, underpinning selective mechanisms, examples of possible empirical evidence, and potential consequences of CFC for selection on males and reproductive outcomes. Note that different mechanisms are not necessarily mutually exclusive. NA indicates that no supporting evidence is currently available.

Recent evidence indicates that genetic benefits of mate choice might be tempered by negative intersexual genetic correlations in fitness caused by intralocus conflict. Thus, adaptive CFC might enable females to ameliorate the costs of intralocus conflict, by optimizing sex allocation based on paternity. In the lizard *Anolis sagrei*, selection on body size is sex specific. Males but not females are selected to be large, and females, which are the heterogametic sex, bias fertilization so that male eggs are preferentially fertilized by the sperm of large males [57]. Furthermore, in the fruit fly *Drosophila simulans*, females discriminate against the sperm from males expressing a sex-ratio distorter [58]. By contrast, the evidence that CFC enables female house mice to avoid fertilization by males carrying the *t* haplotype meiotic driver is less conclusive [59]. Finally, CFC could, in principle, arise as a secondary consequence of viability selection on female fitness, not unlike sensory bias for premating preference evolution. For example, antimicrobial immune response in females might penalize ejaculates with higher microbial loads.

CFC criteria that differ across male–female combinations are often explained by different mechanisms of **genetic compatibility** (Table 1). For example, heterozygosity can be increased when CFC favors male genotypes that are less similar to the female. Broadly consistent with this idea, a recent study in Chinook salmon *Oncorhynchus tshawytscha* found that the sperm–OF interaction predicted embryo survival better than did sperm competitive ability alone, providing evidence of the adaptive role of CFC [43]. In mussels, CFC promotes early embryonic viability in a way that is consistent with egg selection for genetically compatible sperm [41]. These effects could arise because CFC optimizes heterozygosity genome wide or at specific fitness-related loci, such as the MHC (Box 2). The benefits of the former are especially clear when considering inbreeding. In principle, females can avoid inbreeding depression by discriminating against the sperm of close relatives. A powerful approach for examining CFC in this context comes from experimental systems where: (i) females bear a cost of inbreeding depression; and (ii) male sperm competitiveness can be manipulated by experimentally controlling ejaculate size independent of female relatedness. Starting from studies of the field cricket *Gryllus bimaculatus* [60], evidence for CFC against inbreeding is accumulating. Female red junglefowl store fewer sperm following insemination by related than unrelated males (e.g., [11]). AI experiments in guppies showed that CFC biases paternity toward unrelated males in the absence of any premating cues 10, 61. Similarly, *in vitro* sperm selection against sperm from related males has been demonstrated in house mice [9]. More evidence comes from the Mediterranean orb-web spider *Argiope lobata* [62], and the Australian field cricket *Teleogryllus oceanicus* [12]. In other species, however, CFC against inbreeding is either absent or less consistent 25, 63, 64. How can we explain this discrepancy? CFC is more likely to function as inbreeding avoidance under certain conditions, namely: (i) viscous population structure; (ii) intermediate levels of inbreeding depression (promoting male investment in, and female resistance against, inbreeding [11]); and/or (iii) limited opportunities for premating inbreeding avoidance (e.g., due to lack of kin discrimination or male coercion). Alternatively, females might benefit by favoring male genotypes that are similar to the female. Such an assortative pattern is observed in dung flies, where CFC favors males more similar to the female at the phosphoglucomutase (*Pgm*) locus [36], which modulates mobilization of glycogen reserves for flight and temperature-specific larval growth. Similarly, assortative CFC can help prevent hybridization (Box 3).

Box 3 **CFC and Reproductive Isolation**

Rapid coevolution of male and female traits due to postmating sexual selection can lead to postmating-prezygotic (PMPZ) reproductive isolation mediated by competitive or noncompetitive gametic interactions [94]. Just as variation in fertilization success can derive from male (i.e., not CFC) or female (possibly directional CFC) effects or their interaction (nondirectional CFC), PMPZ alone does not automatically implicate CFC (Box 1, main text). In principle, CFC can promote speciation by disfavoring heterospermic fertilization in hybrid zones and secondary contact through conspecific or conpopulation sperm precedence. Evidence for assortative directional CFC in PMPZ isolation is most commonly found in cases of conspecific **sperm precedence** (CSP), in which progeny of females mating with both a heterospecific male and conspecific male are sired predominantly by the conspecific male [94]. Indeed, CSP often occurs in systems where single matings yield viable offspring, and reproductive barriers become evident only under competitive conditions. CSP is thought to arise when divergent selection generates genetic incompatibilities between populations that effectively favor conspecific over heterospecific fertilizations. Although CFC due to genetic incompatibility is considered nondirectional in intraspecific matings, it becomes directional when selection consistently favors conspecific over heterospecific ejaculates.

Although CFC might mediate CSP at any stage from copulation to fertilization (Figure 1B, main text), the earliest and clearest examples come from studies of competitive gamete interactions. For example, variation at the *bindin* and *lysin* loci mediates species-specific fertilization in sea urchins and abalone, respectively, and are under strong positive selection [95]. Furthermore, a highly controlled paired design involving *in vitro* sperm competition between Atlantic salmon and brown trout revealed CSP due to the enhancing effect of OF on conspecific sperm chemoattraction and motility [83]. There is some evidence that CSP mechanisms can also involve earlier stages of CFC as well as multiple mechanisms within a system. CSP in competitive hybrid matings between the crickets *Gryllus campestris* and *Gryllus bimaculatus* is mediated by both preferential storage and a sperm use bias toward conspecific sperm [82]. Moreover, *Drosophila simulans* females use differential ejection and use of alternative sperm storage organs to select against *Drosophila mauritiana* sperm [26].

Beyond generating divergent selection among populations, postcopulatory sexual selection can also affect the establishment and strength of CSP. In house mice, males from populations with high sperm competition outcompeted conpopulation sperm [96], and, in the yellow dung fly, sexually antagonistic coevolution within populations generated heterospecific sperm precedence [97]. Finally, the strength of CSP in mice covaried with the intensity of postmating sexual selection, such that eggs became more discriminatory against heterospecific sperm as the level of sperm competition increased [8].

Alt-text: Box 3

CFC can also result in female costs [65]. Given that CFC might hamper fertilization, females are expected to walk an evolutionary tightrope between the risk of producing offspring with suboptimal sires and reduced fertility. Similarly, responses against the sperm of genetically similar or related males can be constrained by the risk of autoimmunity or immune responses against embryos in viviparous taxa. However, costs of CFC have seldom been quantified [65] (Box 4 ).

**Table I:** Key Challenges for CFC Research.

Proximate	Ultimate
(i) Mechanisms	(ii) Function
Distinguishing female- from male-driven mechanisms	Identifying the adaptive significance of CFC for females
Resolving co-occurrence of multiple CFC mechanisms	Measuring fitness consequences of CFC on males
(ii) Development	(iv) Phylogeny
Characterizing ontogenetic and temporal patterns of variation in CFC	Understanding macroevolutionary patterns of CFC and CFC-related traits

Box 4 **Going Forward: Key Directions for Future CFC Research**

The first 20 years of research have brought us evidence that CFC can occur through several traits and mechanisms, building a platform for future studies of CFC at multiple levels. In keeping with the structure of this review, we use a classic categorization of both proximate and ultimate levels of analysis in biology to identify key challenges, summarized in Table I. Most of the effort so far has focused on categories (i) and (iv).

**Mechanisms**

Studies of mechanisms are arguably the most frequent and best developed of the four categories. However, much remains to be discovered even at this level. Despite recent progress (see main text), unambiguously distinguishing the effect of female- versus male-driven postmating mechanisms remains a key challenge in the study of CFC. Recent work in *Drosophila melanogaster* elucidated the intimate interaction between the effect of inseminated male accessory gland products and the response of the FRT to such effects, including potential for CFC [98]. In addition, it is becoming clear that multiple mechanisms of CFC might occur in the same organism. However, nothing is known about the temporal and spatial scales of these mechanisms and the way they interact with each other to influence paternity. Resolving individual mechanisms of CFC requires investigating more specific mechanisms. For example, in cases where CFC is based on stimuli (e.g., visual or olfactory) of the male phenotype or genotype, future research should determine how these stimuli can trigger a cascade of physiological, neurological, and endocrinological events that cause CFC. Similarly, little is known about mechanisms underpinning CFC when this is triggered by the phenotype of individual sperm cells. Among-sperm variation must exist to allow CFC mechanisms to act, but with few exceptions (e.g., Box 2, main text), such cues remain unidentified, and this area offers a wealth of future investigation. Sperm might convey molecular information to the FRT on which female sperm recognition mechanisms might act (i.e., the molecular sperm passport hypothesis) [46]. For example, the hyaluronic acid receptor CD44 on human sperm is a putative signal of sperm fertilizing potential and, therefore, sperm quality. The FRT, which is rich in hyaluronic acid (e.g., in cervical mucus, OF, and cumulus cells), can discriminate between sperm via the surface expression of CD44, suggesting that the FRT can ‘read’ information available on the sperm surface and accept or reject individual sperm [46]. Although unequivocal evidence is currently lacking, this type of sophisticated sperm discrimination is not unprecedented in other taxonomic groups. As we continue to characterize more mechanisms of CFC, a critical step is to identify the underpinning genetic, physiological, and biochemical processes. Future studies should consider what patterns of gene expression, nucleotide polymorphisms, and proteins explain variation in CFC mechanisms and explore whether metabolic differences within the FRT mediate male × female interactions. The advent of genome-editing tools, such as CRISPR, appear particularly promising, because they allow the surgical deletion or replacement of candidate genes to establish the causal relationships among gene sequence, gene expression, and phenotype.

**Development**

This level of investigation remains almost entirely unexplored, because the majority of CFC work does not consider that patterns of CFC develop or change over the lifespan of a female. However, this is likely in several cases. In honey bees, *Apis mellifera*, the spermathecal fluid of the queen changes in protein composition, suggesting that the first ejaculate initially experiences a biochemical environment considerably different from that experienced by successive inseminations [99]. These ontogenetic changes have intuitive adaptive significance; virgin females might be less selective to reduce the risk that the eggs are not fertilized, and, as matings accumulate, both female choosiness and selectivity can increase. Similarly, CFC mediated by responses of the acquired immune system in vertebrates can change over time, as a female is repeatedly exposed to the sperm of the same male or genotype. Aging might also affect patterns of CFC; for example, in birds, older females can lose sperm from their sperm storage tubules at a faster rate than can younger females.

**Function**

Resolving the adaptive significance of CFC hinges on measuring fitness benefits and costs to females. While some benefits have been explored (see main text), we know next to nothing about the costs of CFC to females. Too-stringent CFC criteria and CFC-driven errors in sperm assessment might result in sperm limitation or enduring unfavorable paternity outcomes. However, there are also likely to be immunological and physiological costs associated with developing and maintaining traits associated with CFC. Understanding how these costs might modulate the intensity and choosiness of CFC and selection on correlated traits is an important area for future research. Experimental evolution represents a powerful multigenerational approach for exploring the potential fitness implications, both costs and benefits, of CFC. This approach also creates the opportunity (and the need) to investigate the (co)evolution of male traits. Understanding how potential costs modulate the intensity and choosiness of CFC is an important area for future research. Given that such costs alter the strength and direction of selection acting on both focal and correlated traits, filling this gap will enhance understanding of CFC at the population level (see below).

**Phylogeny**

Functional studies should also investigate macroevolutionary patterns of CFC-related traits, their coevolution with associated male traits, and the phylogenetic and ecological drivers of such patterns. A comparative approach would also help resolve the role of CFC in reproductive isolation (Box 3, main text) and diversification. Detecting the phylogenetic signature of CFC should be easier than for premating female choice, because CFC can be mediated by morphological or physiological traits that are easier to quantify and compare across species than are more plastic female preference traits. Yet, compared with male reproductive anatomy, female reproductive anatomy is distinctly underrepresented in evolutionary studies, even those investigating CFC! [100].

Alt-text: Box 4

### Evolutionary Consequences for Males

Variance in paternity share generates opportunity for postmating intersexual selection on males (Figure 1A), which can be directional or nondirectional (see above). While the latter is expected to maintain genetic variance and polymorphism, the former is expected to erode additive variance, particularly when directional CFC reinforces patterns of premating female choice (as seen in some of the examples in Figure 2). Conversely, when directional CFC works independently or even against other episodes of sexual selection, opportunity arises for alternative pathways through which males can attain reproductive success via alternative mating tactics. For example, territorial males might invest in traits such as ornaments, armaments, or paternal care that are important in premating sexual selection, while sneakers or satellites might invest in traits that increase fertilizing efficiency after mating, including traits favored by CFC. Alternatively, CFC might bias paternity toward territorial males (e.g., [44]).

Provided that CFC benefits females, there is inescapable **sexual conflict** between the female and the partners whose sperm she disfavors. Therefore, male evolutionary responses to CFC can comprise both strategies that meet female preferences or counteract CFC. Male courtship, mating, and postmating behaviors might ensure that females preferentially use the sperm of one male over those of others [66] and, therefore, can be under selection by CFC. For example, males can prevent or delay female remating through mate guarding, copulatory plugs, or accessory gland proteins that influence female remating behavior. When encountering paternity-biasing traits that allow female control over sperm transfer, males might seek to regain control through derived courtship and mating behaviors (e.g., traumatic insemination) and/or modifications in genital morphology (e.g., [67]). Sperm of some hermaphroditic *Macrostomum* flatworms have evolved bristle-like structures that help prevent them from being sucked out of the female antrum after mating [68]. Patterns of sperm neutralization by females will also influence male strategies of ejaculate expenditure. Males are selected to invest larger ejaculates when females indiscriminately neutralize a fixed number (but not a fixed proportion) of sperm for each insemination. When sperm neutralization is nonrandom and the ejaculates of a male are favored by some females but disfavored by others (nondirectional CFC), males are expected to allocate more when mating in the favored role. However, when individual males are always either favored or disfavored by all females (directional CFC), favored males are expected to always invest less than disfavored males [69]. Other male counter-adaptions can prevent sperm neutralization after mating. Spermatophores of the flatworm *Dugesia gonocephala* [70] and the snail *Helix pomatia* [71] might protect sperm from digestion in the copulatory bursa. In hermaphroditic land snails, love darts transfer an allohormone that delays sperm digestion by stimulating contraction of the copulatory canal, allowing more sperm to be stored [72]. In the heteromorphic *D. pseudoobscura*, nonfertilizing pseudosperm help counter the spermicidal action of the FRT [34]. Similarly, in many taxa, part of the male ejaculate, or even part of the intromittent organ blocks the female genital opening. These traits have been interpreted as defensive adaptations to prevent female remating. A nonmutually exclusive function might be to prevent female sperm ejection.

Finally, if CFC is mediated by immunological responses, males might gain by reducing the bacterial load of their ejaculates. Consistent with this idea, the seminal fluid of several species is enriched with antibodies and other proteins with antimicrobial peptides [73]. Seminal fluid can also contain vesicles, prostasomes, and exosomes with immunosuppressive properties [74], and one of their functions might be to inhibit the female immune response to sperm.

### Male–Female Coevolution

Directional CFC and male responses can drive intersexual coevolution [5], divergence, and speciation (Box 3). Comparative studies have shown phylogenetic signatures of coevolution between FRT morphology and male reproductive traits (e.g., [75]). Genetic correlation between a male trait and CFC for that trait is required for Fisherian runaway selection and has been documented for only a few postmating traits, including in *Onthophagus* dung beetles [76] and *Drosophila* [54] (Figure 2A). These coevolutionary dynamics can often appear to be sexually antagonistic [77]. For example, across waterfowl species, more complex FRTs have evolved in response to male sexual coercion, seemingly to enable females to retain control over paternity, which has in turn driven the evolution of more complex male genitalia [67]. Similarly, egg responses mediating sperm attraction and/or entry (e.g., rapid divergence in signaling proteins and sperm performance–egg defensiveness) have been implicated in coevolution at the gametic level 8, 51, 52.

## Concluding Remarks

The idea of CFC has revolutionized the field of sexual selection by providing a critical counterpoint to male-driven sperm competition and illuminating the potential for female-mediated postmating processes. Evidence accumulated over the past 20 years confirms Eberhard’s [5] intuition that multiple stages between gamete release and fertilization provide opportunities for CFC. However, evaluating this potential requires disentangling male and female effects, something that has been achieved to a degree only in a handful of organisms, and only at some of these stages. This is because the intimate correspondence of male stimuli and female responses that characterizes the cascade of events from insemination to fertilization often means that the very notion of disentangling male and female effects can be a misleadingly simplistic dichotomy.

If demonstrating CFC is difficult, understanding its functional significance is similarly challenging. Despite intense effort, evidence that polyandry and CFC benefit females remains remarkably elusive 78, 79. One reason for this is that multiple hypotheses have been proposed to explain the adaptive significance of CFC, and the multitude of these hypotheses makes it difficult to rule out the null hypothesis. Furthermore, adaptive CFC is likely driven by genetic benefits to the offspring, which are typically small [78]. We predict that CFC can have an important role under specific conditions, namely in highly polyandrous species, where premating female choice is difficult or severely constrained, such as broadcast spawners or internal fertilizers, where males can coerce females into mating. Investigations of such mating systems have been promising and suggest that, here, CFC can be an agent of evolutionary exaggeration and diversification through its role in sexual selection on males and intersexual coevolutionary dynamics.

## Glossary

**Directional/nondirectional selection**: in the context of female choice, males are under directional selection when female preferences are shared, and nondirectional selection when female preferences differ among individuals.
**Egg defensiveness**: egg resistance to fertilization driven by selection for increased sperm fertilizing potential via sperm competition, increasing risk of polyspermy.
**Female reproductive fluids**: substances released by different regions of the female reproductive tract (FRT) or associated accessory glands, commonly called ‘egg water’ in invertebrates and ‘ovarian fluid’ (OF) in vertebrates.
**Fisherian runaway**: a scenario in which linkage disequilibrium between female preference and male ornament, genes generated by assortative mating, can lead to unstable exaggerations of both traits, favored by the reproductive advantage of the offspring.
**Genetic compatibility hypothesis**: cryptic female choice (CFC) allows females to minimize the risk of fertilizations by sperm that have haplotypes that are not compatible with their own genome.
**Good genes**: models of sexual selection that assume that extreme ornaments indicate the genetic quality of the bearer (usually males), defined as breeding value for fitness.
**‘Good sperm’ hypothesis**: predicts that females mate polyandrously to ensure that males of high genetic quality, and increased competitive fertilization success, sire their offspring.
**P_2_**: the paternity and/or fertilization success of the second of two males to copulate with a female, expressed as a proportion of total offspring and/or egg number.
**Polyandry**: mating systems in which females mate with more than one sexual partner. In the context of this review, we focus on situations in which polyandry creates the opportunity for the sperm of different males to co-occur for the fertilization of the same set of ova.
**Polyspermy**: the condition where multiple sperm enter the egg during fertilization (described as pathological when polyspermy has fatal consequences for the zygote)
**S_2_**: the proportion of stored sperm from a second of two males to mate relative to the total sperm.
**Sexual conflict**: a divergence in the fitness interests of males and females over reproductive decisions or the outcome of their reproductive interactions.
**Sexual selection**: selection for traits conferring an advantage in intrasexual competition over mating and fertilization; occurs both before (premating), during, and after (postmating) copulation, either as competition between members of the same sex (usually males) for access to mates (intrasexual), or when members of one sex (usually females) choose individuals and/or sperm of the opposite sex (intersexual).
**‘Sexy sperm’ hypothesis**: predicts linkage disequilibrium between female polyandry and male sperm competitive efficiency leading to polyandrous females having a selective advantage over monandrous females, because their sons will be sired by males with competitively superior sperm and will inherit this trait.
**Sperm chemotaxis**: the movement of sperm toward eggs, following a gradient of chemoattractants released by unfertilized eggs.
**Sperm competition**: occurs when the ejaculates of two or more males compete to fertilize the eggs of a female.
**Sperm precedence**: when the sperm of one male has an advantage over another male, for example by being preferentially selected by the female during mating.

## References

[1] Darwin C.R. (1871). The Descent of Man and Selection in Relation to Sex.

[2] Parker G.A. (1970). Sperm competition and its evolutionary consequences in the insects. Biol. Rev..

[3] Childress D., Hartl D.L. (1972). Sperm preference in *Drosophila melanogaster*. Genetics.

[4] Thornhill R. (1983). Cryptic female choice and its implications in the scorpionfly *Harpobittacus nigriceps*. Am. Nat..

[5] Eberhard W.G. (1996). Female Control: Sexual Selection by Cryptic Female Choice.

[6] Arnqvist G., Shuker D., Simmons L.W. (2014). Cryptic female choice. The Evolution of Insect Mating Systems.

[7] Pilastro A. (2004). Cryptic female preference for colorful males in guppies. Evolution.

[8] Martin-Coello J. (2009). Sperm competition promotes asymmetries in reproductive barriers between closely related species. Evolution.

[9] Firman R.C., Simmons L.W. (2015). Gametic interactions promote inbreeding avoidance in house mice. Ecol. Lett..

[10] Gasparini C., Pilastro A. (2011). Cryptic female preference for genetically unrelated males is mediated by the ovarian fluid in the guppy. Proc. R. Soc. London B.

[11] Løvlie H. (2013). Cryptic female choice favours sperm from major histo-compaibility complex-dissimilar males. Proc. R. Soc. London B.

[12] Tuni C. (2013). Female crickets assess relatedness during mate guarding and bias storage of sperm towards unrelated males. J. Evol. Biol..

[13] Pattarini J.M. (2006). Mechanisms underlying the sperm quality advantage in *Drosophila melanogaster*. Evolution.

[14] Bennison C. (2015). Long sperm fertilize more eggs in a bird. Proc. R. Soc. London B.

[15] Holman L. (2011). Random sperm use and genetic effects on worker caste fate in *Atta colombica* leaf-cutting ants. Mol. Ecol..

[16] Bretman A. (2009). Promiscuous females avoid inbreeding by controlling sperm storage. Mol. Ecol..

[17] Lymbery R.A. (2016). Fluorescent sperm offer a method for tracking the real-time success of ejaculates when they compete to fertilise eggs. Sci. Rep..

[18] Manier M.K. (2010). Resolving mechanisms of competitive fertilization success in *Drosophila melanogaster*. Science.

[19] Lüpold S. (2013). Female mediation of competitive fertilization success in *Drosophila melanogaster*. Proc. Natl. Acad. Sci. U. S. A..

[20] Ala-Honkola O., Manier M.K. (2016). Multiple mechanisms of cryptic female choice act on intraspecific male variation in *Drosophila simulans*. Behav. Ecol. Sociobiol..

[21] Droge-Young E.M. (2016). Resolving mechanisms of short-term competitive fertilization success in the red flour beetle. J. Insect Physiol..

[22] Xu J., Wang Q. (2010). Mechanisms of last male precedence in a moth: sperm displacement at ejaculation and storage sites. Behav. Ecol..

[23] Herberstein M.E. (2011). Sperm storage and copulation duration in a sexually cannibalistic spider. J. Ethol..

[24] Pilastro A. (2007). Copulation duration, insemination efficiency and male attractiveness in guppies. Anim. Behav..

[25] Ala-Honkola O. (2011). No evidence for postcopulatory inbreeding avoidance in *Drosophila melanogaster*. Evolution.

[26] Manier M.K. (2013). Postcopulatory sexual selection generates speciation phenotypes in *Drosophila*. Curr. Biol..

[27] Peretti A.V., Eberhard W.G. (2010). Cryptic female choice via sperm dumping favours male copulatory courtship in a spider. J. Evol. Biol..

[28] Pizzari T., Birkhead T.R. (2000). Female feral fowl eject sperm of subdominant males. Nature.

[29] Dean R. (2011). The risk and intensity of sperm ejection in female birds. Am. Nat..

[30] Wagner R.H. (2004). Female choice of young sperm in a genetically monogamous bird. Proc. R. Soc. London B.

[31] Davies N.B. (1983). Polyandry, cloaca-pecking and sperm competition in dunnocks. Nature.

[32] Friesen C.R. (2016). Female behaviour and the interaction of male and female genital traits mediate sperm transfer during mating. J. Evol. Biol..

[33] Troisi A., Carosi M. (1998). Female orgasm rate increases with male dominance in Japanese macaques. Anim. Behav..

[34] Holman L., Snook R. (2008). A sterile sperm caste protects brother fertile sperm from female-mediated death in *Drosophila pseudoobscura*. Curr. Biol..

[35] den Boer S.P.A. (2010). Seminal fluid mediates ejaculate competition in social insects. Science.

[36] Ward P.I. (2000). Cryptic female choice in the yellow dung fly *Scathophaga stercoraria* (L.). Evolution.

[37] Miller G.T., Pitnick S. (2002). Sperm-female coevolution in *Drosophila*. Science.

[38] Lüpold S. (2012). How multivariate ejaculate traits determine competitive fertilization success in *Drosophila melanogaster*. Curr. Biol..

[39] Snook R.R., Hosken D.J. (2004). Sperm death and dumping in *Drosophila*. Nature.

[40] Barnett M. (1995). Female mediation of sperm competition in the millipede *Alloporus uncinatus* (Diplopoda: Spirostreptidae). Behav. Ecol. Sociobiol..

[41] Oliver M., Evans J.P. (2014). Chemically moderated gamete preferences predict offspring fitness in a boradcast spawner. Proc. R. Soc.London B.

[42] Urbach D. (2005). Effects of ovarian fluid on sperm velocity in Arctic charr (*Salvelinus alpinus*). Behav. Ecol. Sociobiol..

[43] Rosengrave P. (2016). Cryptic female choice enhances fertilization success and embryo survival in chinook salmon. Proc. R. Soc. London B.

[44] Alonzo S.H. (2016). Ovarian fluid allows directional cryptic female choice despite external fertilization. Nat. Commun..

[45] Herberstein M.E. (2011). Sperm dynamics in spiders. Behav. Ecol..

[46] Holt W.V., Fazeli A. (2015). Do sperm possess a molecular passport? Mechanistic insights into sperm selection in the female reproductive tract. Mol. Hum. Reprod..

[47] Stapper A.P. (2015). Assortative mating drives linkage disequilibrium between sperm and egg recognition protein loci in the sea urchin *Strongylocentrotus purpuratus*. Mol. Biol. Evol..

[48] Kamei N., Glabe C.G. (2003). The species-specific egg receptor for sea urchin sperm adhesion is EBR1, a novel ADAMTS protein. Genes Develop..

[49] Ghaderi D. (2011). Sexual selection by female immunity against paternal antigens can fix loss of function alleles. Proc. Natl. Acad. Sci..

[50] Rulicke T. (1998). MHC-genotype of progeny influenced by parental infection. Proc. R. Soc. London B.

[51] Levitan D.R. (2007). The risk of polyspermy in three congeneric sea urchins and its implications for gametic incompatibility and reproductive isolation. Evolution.

[52] Firman R.C. (2014). The coevolution of ova defensiveness with sperm competitiveness in house mice. Am. Nat..

[53] Firman R.C., Simmons L.W. (2013). Sperm competition risk generates phenotypic plasticity in ovum fertilizability. Proc. R. Soc. London B.

[54] Lüpold S. (2016). How sexual selection can drive the evolution of costly sperm ornamentation. Nature.

[55] Yasui Y. (1997). A ‘good-sperm’ model can explain the evolution of costly multiple mating by females. Am. Nat..

[56] Curtsinger J.W. (1991). Sperm competition and the evolution of multiple mating. Am. Nat..

[57] Calsbeek R., Bonneaud C. (2008). Postcopulatory fertilization bias as a form of cryptic sexual selection. Evolution.

[58] Angelard C. (2008). Female-driven mechanisms, ejaculate size and quality contribute to the lower fertility of sex-ratio distorter males in *Drosophila simulans*. BMC Evol. Biol..

[59] Sutter A., Lindholm A.K. (2016). No evidence for female discrimination against male house mice carrying a selfish genetic element. Curr. Zool..

[60] Tregenza T., Wedell N. (2002). Polyandrous females avoid costs of inbreeding. Nature.

[61] Fitzpatrick J.L. (2014). Male–female relatedness and male reproductive investment in guppies. Biol. Lett..

[62] Welke K., Schneider J.M. (2009). Inbreeding avoidance through cryptic female choice in the cannibalistic orb-web spider *Argiope lobata*. Behav. Ecol..

[63] Mack P.D. (2002). Sperm competitive ability and genetic relatedness in *Drosophila melanogaster*: similarity breeds contempt. Evolution.

[64] Denk A.G. (2005). Paternity in mallards: effects of sperm quality and female sperm selection for inbreeding avoidance. Behav. Ecol..

[65] Ward P.I. (2008). A cost of cryptic female choice in the yellow dung fly. Genetica.

[66] Schneider J.M., Lesmono K. (2009). Courtship raises male fertilization success through post-mating sexual selection in a spider. Proc. R. Soc. London B.

[67] Brennan P.L.R. (2007). Coevolution of male and female genital morphology in waterfowl. PLoS One.

[68] Schärer L. (2011). Mating behavior and the evolution of sperm design. Proc. Natl. Aca. Sci. U. S. A..

[69] Parker G.A., Pizzari T. (2010). Sperm competition and ejaculate economics. Biol. Rev. Camb. Philos. Soc..

[70] Lind H. (1973). The functional significance of the spermatophore and the fate of spermatozoa in the genital tract of *Helix pomatia* (Gastropoda: Stylommatophora). J. Zool..

[71] Vreys C. (1997). Formation, transfer and assimilation of the spermatophore of the hermaphroditic planarian *Dugesia gonocephala* (Tricladida, Paludicola). Can. J. Zool..

[72] Chase R., Blanchard K.C. (2006). The snail’s love-dart delivers mucus to increase paternity. Proc. R. Soc. London B.

[73] Peng Y. (2016). Seminal fluid of honeybees contains multiple mechanisms to combat infections of the sexually transmitted pathogen *Nosema apis*. Proc. R. Soc. London B.

[74] Vojtech L. (2014). Exosomes in human semen carry a distinctive repertoire of small non-coding RNAs with potential regulatory functions. Nucelic Acids Res..

[75] Higginson D.M. (2012). Female reproductive tract form drives the evolution of complex sperm morphology. Proc. Natl. Acad. Sci. U. S. A..

[76] Simmons L.W., Kotiaho J.S. (2007). Quantitative genetic correlation between trait and preference supports a sexually selected sperm process. Proc. Natl. Acad. Sci. U. S. A..

[77] Rowe L., Arnqvist G. (2012). Sexual selection and the evolution of genital shape and complexity in water striders. Evolution.

[78] Slatyer R.A. (2012). Estimating genetic benefits of polyandry from experimental studies: a meta-analysis. Biol. Rev..

[79] Lumley A.J. (2016). Post-copulatory oppoertunities for sperm competition and cryptic female choice provide no offspring benefits in externally fertilizing salmon. Open Sci..

[80] Cordoba-Aguilar A. (1999). Male copulatory sensory stimulation induces female ejection of rival sperm in a damselfly. Proc. R. Soc. London B.

[81] Fisher D.O. (2006). Post-mating sexual selection increase lifetime fitness of polyandrous females in the wild. Nature.

[82] Tyler F. (2013). Multiple post-mating barriers to hybridization in field crickets. Mol. Ecol..

[83] Yeates S.E. (2013). Cryptic choice of conspecific sperm controlled by the impact of ovarian fluid on sperm swimming behavior. Evolution.

[84] Birkhead T.R. (2000). Defining and demonstrating postcopulatory female choice – again. Evolution.

[85] Bjork A. (2007). Adaptive modulation of sperm production rate in *Drosophila bifurca*, a species with giant sperm. Biol. Lett..

[86] Parker G.A. (1990). Analysing sperm competition data: simple models for predicting mechanisms. Behav. Ecol. Sociobiol..

[87] Eggert A.K. (2003). Linear models for assessing mechanisms of sperm competition: the trouble with transformations. Evolution.

[88] Neff B.D., Wahl L.M. (2004). Mechanisms of sperm competition: testing the fair raffle. Evolution.

[89] Manier M.K. (2013). An analytical framework for estimating fertilization bias and the fertilization set from multiple sperm-storage organs. Am. Nat..

[90] Birkhead T.R. (2004). Nontransitivity of paternity in a bird. Evolution.

[91] Milinski M. (2006). The major histocompatibility complex, sexual selection, and mate choice. Ann. Rev. Ecol. Evol. Syst..

[92] Yeates S.E. (2009). Atlantic salmon eggs favour sperm in copetition that have similar major histocompatibility alleles. Proc. R. Soc. London B.

[93] Gasparini C. (2015). Major histocompatibility complex similarity and sexual selection: different does not always mean attractive. Mol. Ecol..

[94] Howard D.J., Birkhead T.R. (2009). Sperm and speciation. Sperm Biology: An Evolutionary Perspective.

[95] Swanson W.J., Vacquier V.D. (2002). Reproductive protein evolution. Ann. Rev. Ecol. Syst..

[96] Firman R.C., Simmons L.W. (2014). No evidence of conpopulation sperm precedence between allopatric populations of house mice. PLoS One.

[97] Hosken D.J. (2002). Heteropopulation males have a fertilization advantage during sperm competition in the yellow dung fly (*Scathophaga stercoraria*). Proc. R. Soc. London B.

[98] Sirot L.K., Wolfner M.F., Peretti A.V., Aisenberg A. (2015). Who’s zooming who? Seminal fluids and cryptic female choice in Diptera. Cryptic Female Choice in Arthropods. Patterns, Mechanisms and Prospects.

[99] Baer B., Peretti A.V., Aisenberg A. (2015). Female choice in social insects. Cryptic Female Choice in Arthropods. Patterns, Mechanisms and Prospects.

[100] Ah-King M. (2014). Genital evolution: why are females still understudied?. PLoS Biol..

