# Supplementary material for: Postmating Female Control: 20 Years of Cryptic Female Choice
Source: Trends Ecol Evol. 2017 May;32(5):368–82. doi: 10.1016/j.tree.2017.02.010 (PMC5511330; doi:10.1016/j.tree.2017.02.010)
Supplement: Supplementary file 1 [file mmc1.docx]

Box 4: Going forward: Key directions for future CFC research

The first 20 years of research have brought us evidence that Cryptic Female Choice (CFC) can occur through a number of traits and mechanisms, building a platform for future studies of CFC at multiple levels. In keeping with the structure of this review, we use a classic categorization of both proximate and ultimate levels of analysis in biology to identify key challenges, summarized in the table below. Most of the effort so far has focused on categories (1) and (4).


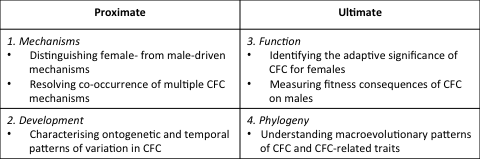


*Mechanisms*. Studies of mechanisms are arguably the most frequent and best developed of the four categories. However, much remains to be discovered even at this level. Despite recent progress (see main text), unambiguously distinguishing the effect of female- vs male-driven postmating mechanisms remains a key challenge in the study of CFC. Recent work in *D. melanogaster* has elucidated the intimate interaction between the effect of inseminated male accessory gland products and the response of the FRT to such effects, including potential for CFC [98]. In addition, it is becoming clear that multiple mechanisms of CFC might occur in the same organism. However, nothing is known about the temporal and spatial scales of these mechanisms and the way they interact with each other to influence paternity. Resolving individual mechanisms of CFC requires investigating more specific mechanisms. For example, in cases where CFC is based on stimuli (e.g., visual or olfactory) of the male phenotype or genotype, future research should determine how these stimuli can trigger a cascade of physiological, neurological, and endocrinological events that cause CFC. Similarly, very little is known about mechanisms underpinning CFC when this is triggered by the phenotype of individual sperm cells. Among-sperm variation must exist to allow CFC mechanisms to act, but with few exceptions (e.g., Box 2), such cues remain unidentified, and this area offers a wealth of future investigation. Sperm might convey molecular information to the FRT on which female sperm recognition mechanisms might act (“molecular sperm passport hypothesis”) [46]. For example, the hyaluronic acid receptor CD44 on human sperm is a putative signal of sperm fertilizing potential and therefore sperm quality. The FRT, which is rich in hyaluronic acid (e.g., in cervical mucus, OF, cumulus cells), can discriminate between sperm via the surface expression of CD44, suggesting that the FRT can “read” information available on the sperm surface and accept or reject individual sperm [46]. Although unequivocal evidence is currently lacking, this type of sophisticated sperm discrimination is not unprecedented in other taxonomic groups. As we continue to characterize more mechanisms of CFC, a critical step is to identify the underpinning genetic, physiological, and biochemical processes. Future studies should consider what patterns of gene expression, nucleotide polymorphisms, and proteins explain variation in CFC mechanisms and explore whether metabolic differences within the FRT mediate male×female interactions. The advent of genome editing tools such as CRISPR appear particularly promising, as they allow the surgical deletion or replacement of candidate genes, to establish the causal relationships among gene sequence, gene expression, and phenotype.

*Development.* This level of investigation remains almost entirely unexplored, as the majority of CFC work does not consider that patterns of CFC develop or change over the lifespan of a female. This is, however, quite likely in several cases. In honey bees, *Apis mellifera*, the queen's spermathecal fluid changes in protein composition, suggesting that the first ejaculate might initially experience a biochemical environment considerably different from that experienced by successive inseminations [99]. These ontogenetic changes have intuitive adaptive significance; virgin females might be less selective in order to reduce the risk that the eggs are not fertilized, and as matings accumulate, both female choosiness and selectivity can increase. Similarly, CFC mediated by responses of the acquired immune system in vertebrates can change over time, as a female is repeatedly exposed to the sperm of the same male or genotype. Ageing might also affect patterns of CFC; e.g. in birds, older females can lose sperm from their sperm storage tubules at a faster rate than younger females.

*Function.* Resolving the adaptive significance of CFC hinges on measuring fitness benefits and costs to females. While some benefits have been explored (see main text), we know next to nothing about the costs of CFC to females. Too stringent CFC criteria and CFC-driven errors in sperm assessment might result in sperm limitation or enduring unfavorable paternity outcomes. However, there are also likely to be immunological and physiological costs associated with developing and maintaining traits associated with CFC. Understanding how these costs might modulate the intensity and choosiness of CFC and selection on correlated traits is an important area for future research. Experimental evolution represents a powerful multi-generational approach for exploring the potential fitness implications, both costs and benefits, of CFC. This approach also creates the opportunity (and the need) to investigate the (co)evolution of male traits. Understanding how potential costs modulate the intensity and choosiness of CFC is an important area for future research. As such costs alter the strength and direction of selection acting on both focal and correlated traits, filling this gap will enhance understanding of CFC at the population level (see below).

*Phylogeny.* Functional studies should also investigate macroevolutionary patterns of CFC-related traits, their co-evolution with associated male traits, and the phylogenetic and ecological drivers of such patterns. A comparative approach would also help resolve the role of CFC in reproductive isolation (Box 3) and diversification. Detecting the phylogenetic signature of CFC should be easier than for premating female choice, because CFC can be mediated by morphological or physiological traits that are easier to quantify and compare across species than more plastic female preference traits. Yet, compared to male reproductive anatomy, female reproductive anatomy is distinctly underrepresented in evolutionary studies, even those investigating CFC! [100].
